# CME Programming Improved Knowledge of Biomarker and Therapeutic Data in Alzheimer’s Disease From a 2024 Dementia Conference Among Neurologists and Primary Care Physicians

**DOI:** 10.1002/alz70859_099477

**Published:** 2025-12-25

**Authors:** Thomas Finnegan, Frances McFarland, Austin Baiardi, Ronald Petersen, Philip Scheltens

**Affiliations:** ^1^ Medscape Education, Newtown Square, PA USA; ^2^ Medscape Education, Newark, NJ USA; ^3^ Mayo Clinic Alzheimer's Disease Research Center, Rochester, MN USA; ^4^ Alzheimer Center Amsterdam, Neurology, Vrije Universiteit Amsterdam, Amsterdam UMC location VUmc, Amsterdam Netherlands

## Abstract

**Background:**

The last several decades have seen intense research into Alzheimer’s disease (AD) resulting in several current and emerging disease modifying treatments that target hallmarks of the AD pathology. Previous evidence has indicated that neurologists and primary care physicians (PCPs) frequently lack knowledge of the most recent findings pertaining to the use of biomarkers and antibody‐based therapies in AD. An online CME program was developed to educate neurologists and PCPs on biomarker and therapeutic data in AD from a 2024 dementia conference.

**Method:**

The online CME activity consisted of a chapterized 30‐minute video presentation featuring dementia specialists who reviewed data from a 2024 dementia conference on biomarkers or therapeutics. Educational effect was assessed by comparing a matched sample of responses to four identical questions presented before and directly after exposure to the intervention. A paired samples t‐test assessed the number of correct responses and for confidence rating; McNemar’s test was used to identify significant differences between pre‐ and post‐assessment question responses. Cohen’s d was used to calculate the effect size of the online education. Data were collected between August 2024 and October 2024.

**Result:**

A moderate educational effect was observed for both neurologist (*n*=88; *d*=.77, *p*<0.001) and PCP (*n*=141; *d*=.75, *p*<.001) learners. Both physician groups demonstrated significant (P <.05) pre‐ vs post‐educational improvements on the following: clinical data on AD diagnostic accuracy comparing blood‐based biomarker testing to a clinical diagnosis made by a physician and risk factors for amyloid‐relating imaging abnormalities associated with an approved anti‐amyloid therapy (AAT). PCPs, but not neurologists, demonstrated a pre‐post educational improvement on a question about outcomes from an open‐label extension study of an approved ATT. Participation in the activity resulted in 44% of neurologists and 48% of PCPs reporting an increase in confidence regarding their ability to apply the latest clinical data on approved AATs in clinical practice.

**Conclusion:**

The results indicated that a CME‐certified 30‐minute video program was effective at improving knowledge among a diversity of physician learners regarding emerging data from a recent medical conference. Future CME should continue to highlight impactful new data released at medical conferences.

